# Editorial: Vision in Cephalopods: Part II

**DOI:** 10.3389/fphys.2021.731780

**Published:** 2021-08-02

**Authors:** Frederike D. Hanke, Chuan-Chin Chiao, Daniel C. Osorio

**Affiliations:** ^1^Institute for Biosciences, Neuroethology, University of Rostock, Rostock, Germany; ^2^Department of Life Science, National Tsing Hua University, Hsinchu, Taiwan; ^3^School of Life Sciences, University of Sussex, Brighton, United Kingdom

**Keywords:** eye, optics, visual system, visual adaptations, octopus, sepia

Coleoid cephalopods are much like fish, with single chambered eyes, large visual brain areas, and complex behaviors, but they have evolved independently, and their locomotion—inspiration to the field of soft robotics (Calisti et al., 2011; Kim et al., 2013), adaptive coloration, and polarization vision are quite unlike those of vertebrates (Hochner et al., 2006; Hanlon and Messenger, 2018). What then do these fascinating molluscs, often said to be intelligent, reveal about chance and necessity in the evolution of brains and behavior?

Our 2018 Frontiers in Physiology Research Topic “Vision in cephalopods” (https://www.frontiersin.org/research-topics/4856/vision-in-cephalopods) showed that cephalopod vision research is a small but flourishing field. The present collection of eight research articles and one review demonstrates the strength and significance of the field, encompassing subjects such as phototransduction (Bonadè et al.), psychophysics of polarization vision (Nahmad-Rohen and Vorobyev), visual development (Groeger et al.) and two of the most distinctive cephalopod behaviors—prey capture (Wu et al.; Brauckhoff et al.) and adaptive coloration (Hadjisolomou et al.). Cephalopods are best known in neuroscience for the squid giant axon and octopus cognition, but the present collection finds cuttlefish of the genus *Sepia* as the subject of all but two on *Octopus* and one on a squid.

Most cephalopods are color blind (but c.f. Stubbs and Stubbs, 2016), but polarization vision might substitute color vision (Pignatelli et al., 2011), allowing them to judge surface properties, and to mitigate the effects of scatter in turbid water. However, whereas most animals process color and luminance in separate visual pathways, Nahmad-Rohen and Vorobyev find that octopus use the same system for polarization, and luminance. Polarization patterns—which are invisible to the human eye—feature in the repertoire of visual cephalopod communication signals. Here, López Galán et al. highlight the richness of these signals, and the dynamics in courtship displays of the cuttlefish *Sepia plangon*, which has 57 body pattern components deployed in 18 body patterns. Many of these patterns are displayed only briefly, and an attempt to test these small cuttlefish with 3D printed models of conspecifics failed because the models lacked the dynamics of the visual signals. It would be interesting to know how far learning and motor skill play a part in the function of these elaborate visual signals as they do in bird song (Marler, 1990). Dynamic patterns are possible because cephalopods' color change is mediated by chromatophores, which are directly innervated by motoneurons (Messenger, 2001), allowing rapid change and the production of moving patterns known as passing cloud displays. Here Hadjisolomou et al. show that individual chromatophores of the squid *Doryteuthis pealeii* can respond to a flash with a mean latency of only 50 ms. Visual movement is also important in prey capture when both prey and predator move, and Wu et al. find that the cuttlefish *Sepia pharaonis* can extract the speed and direction from their moving prey to track prey and to select the visual hunting strategy most appropriate for the specific situation.

Turning to visual ecology, Goerger et al. investigate how turbidity affects visual development of the cuttlefish *Sepia officinalis*; surprisingly a low level of turbidity during larval development improves polarization sensitivity. Cephalopods also have to cope with changes in ambient luminance. The common octopus *Octopus vulgaris* can adapt to sudden changes in luminance with a rapid pupillary response (Soto et al.). With the characterization of the dynamics of the pupil of *Octopus vulgaris*, our understanding of vision in this cephalopod species, that is/has been widely used in visual (discrimination) experiments, was advanced (for review see Hanke and Kelber). While a mobile pupil can be of advantage in an inhomogeneous light environment, ambient luminance also changes with a daily cycle. Although some cephalopod species are active during the day, Brauckhoff et al. show that the cuttlefish can hunt in dim light conditions but not in complete darkness.

Where next? In the 1930's, Young (1938) already highlighted the potential of *Octopus* for neuroscience leading to wonderful anatomical work, behavioral studies, and began investigations of the squid giant axon, but nevertheless research on “simple” nervous systems mostly focused on insects and gastropod molluscs. Modern physiological methods offer the potential for recording from cephalopod brain to understand the visual motor control circuitry, learning and more. We are unlikely to attract the support offered to key model organisms or for clinical applications, but, as Young already realized, cephalopods offer unique insight into principles of sensory-motor control, cognition and evolutionary neuroscience that are of the widest significance.

## Author Contributions

All authors listed have made a substantial, direct and intellectual contribution to the work, and approved it for publication.

## Conflict of Interest

The authors declare that the research was conducted in the absence of any commercial or financial relationships that could be construed as a potential conflict of interest.

## Publisher's Note

All claims expressed in this article are solely those of the authors and do not necessarily represent those of their affiliated organizations, or those of the publisher, the editors and the reviewers. Any product that may be evaluated in this article, or claim that may be made by its manufacturer, is not guaranteed or endorsed by the publisher.
